# Preclinical Ocular Pharmacokinetics and Efficacy of Novel Tivozanib Eye Drops for Neovascular Age-Related Macular Degeneration

**DOI:** 10.1167/iovs.67.8.55

**Published:** 2026-07-27

**Authors:** Kyosuke Satake, Shoko Koshiba, Hiroki Haniuda, Toru Amano, Masanori Hiura, Toshihiko Ishii, Shinya Horita

**Affiliations:** 1 Research Division, Kyowa Kirin Co., Ltd. Sunto-gun, Shizuoka, Japan; 2 Manufacturing Division, Kyowa Kirin Co., Ltd. Sunto-gun, Shizuoka, Japan; 3 Development Division, Kyowa Kirin Co., Ltd. Sunto-gun, Shizuoka, Japan; 4 Development Division, Kyowa Kirin North America, Princeton, New Jersey, United States

**Keywords:** nanocrystallization, tivozanib, eye drop formulation, neovascular age-related macular degeneration (nAMD), ocular pharmacokinetics

## Abstract

**Purpose:**

In neovascular age-related macular degeneration (nAMD), invasive intravitreal injection of anti-vascular endothelial growth factor (VEGF) drugs is the current standard of care, highlighting the need for noninvasive eye drop formulations. We aimed to assess the ocular pharmacokinetics and anti-angiogenic efficacy of nanocrystallized tivozanib (nTivo) eye drops in rabbits and monkeys.

**Methods:**

We investigated the ocular distribution of nTivo eye drops and conventional microcrystallized tivozanib eye drops in albino and pigmented rabbits. The anti-angiogenic efficacy of nTivo eye drops was evaluated in laser-induced choroidal neovascularization (CNV) model monkeys.

**Results:**

In pigmented rabbits, nTivo eye drops showed up to 9.5-fold higher drug delivery efficiency to the retina/choroid relative to microcrystal formulations, indicating that nanocrystallization enhances the drug delivery efficiency of tivozanib to posterior eye tissues. Additionally, accumulation of the ocular exposure was observed with the repeated instillation of nTivo eye drops, and the elimination half-life (t_1/2_) of tivozanib was longer in the retina/choroid (247.5 hours) than in serum (49.7 hours). In comparison to albino rabbits, pigmented rabbits showed higher exposure (3.2-fold) and longer t_1/2_ (5.7-fold) in the retina/choroid, suggesting that the melanin binding properties of tivozanib potentially contribute to its accumulation and prolonged retention in the retina and choroid. In the monkey CNV model, nTivo eye drops significantly reduced the neovascularization lesion area.

**Conclusions:**

The nTivo eye drop formulation may be a potential new treatment option for nAMD due to its preferable ocular pharmacokinetic and anti-angiogenic profiles based on potential contributions of nanocrystallization and the melanin binding properties of tivozanib.

Age-related macular degeneration (AMD) is a chronic disease that affects the macular area of the posterior segment of the eye and is the leading cause of severe vision impairment and legal blindness, particularly among individuals >55 years of age.^1^^–^^3^ It is estimated that approximately 200 million people were affected by AMD globally in 2020, and the number is projected to increase to approximately 288 million by 2040.^4^

Vascular endothelial growth factor (VEGF) is a critical regulator of neovascularization and plays a pivotal role in the pathophysiology of neovascular AMD (nAMD), which is a subclass of AMD.^5^^,^^6^ Anti-VEGF therapies, including ranibizumab, aflibercept, brolucizumab, and faricimab, have demonstrated substantial efficacy in preserving and even improving vision in patients with nAMD.^7^^–^^14^ However, these therapies require intravitreal injections, which impose significant burdens on patients due to their invasive nature and frequency of administration.^15^ Considering these obstacles, there is a clear medical need for alternative noninvasive, convenient, and effective treatment options for nAMD that can alleviate the patient burden.

Eye drop formulations are preferred due to their noninvasive nature and reduced treatment burden in comparison to current intravitreal injection therapies.^16^^,^^17^ Several eye drop products including small molecule VEGF receptor inhibitors (e.g., pazopanib, regorafenib, LHA510, and PAN-90806) have been investigated for nAMD treatment applications.^18^^–^^23^ However, the development of most of these products was discontinued due to insufficient clinical efficacy. These findings suggest that inadequate drug exposure in the posterior segment of the eye is a major issue underlying the insufficient clinical efficacy in nAMD.^24^

Nanocrystallization has been investigated due to its potential to enhance ocular drug delivery of eye drops to posterior eye tissues. In contrast to conventional eye drop formulations with micro-size crystals, nanocrystallization enlarges the surface area of particles with the same amounts of active ingredients, resulting in a higher dissolution rate and increased apparent drug solubility.^25^ Nanocrystallization is expected to enhance the drug delivery of eye drop products to the posterior segment of the eye.

Tivozanib is a small molecule tyrosine kinase inhibitor that strongly and selectively inhibits phosphorylation of three types of VEGF receptors (VEGFRs; VEGFR-1–3).^26^ The oral formulation of tivozanib (FOTIVDA) has been approved by the US Food and Drug Administration (FDA) and the European Medicines Agency (EMA) for advanced renal cell carcinoma.^27^^,^^28^ We have developed a novel eye drop formulation of nanocrystallized tivozanib (nTivo) for the treatment of nAMD.

We aimed to assess the ocular pharmacokinetics of nTivo eye drops in rabbits and monkeys, and its anti-angiogenic efficacy in laser-induced choroidal neovascularization (CNV) model monkeys.

## Methods

### Chemicals

Tivozanib was obtained from AVEO Oncology (Boston, MA, USA). KRN633 (tivozanib derivative) and ^13^C, ^2^H-labeled tivozanib were obtained from Kyowa Kirin Co., Ltd. (Tokyo, Japan). Water for injection and physiological saline were purchased from Otsuka Pharmaceutical Factory, Inc. (Tokushima, Japan). Acetonitrile, methanol, formic acid, and ammonium acetate were purchased from FUJIFILM Wako Pure Chemical Corporation (Osaka, Japan). Thiopental sodium solution (Ravonal 0.5*g* for injection) was purchased from Nipro ES Pharma Co., Ltd. (Osaka, Japan). All chemicals were of high purity or reagent grade.

### Test Article

Tivozanib bulk powder and dispersion medium were transferred into a milling chamber of DYNO-MILL ECM-AP 05 (Willy A. Bachofen AG) with yttrium-stabilized zirconia milling beads. The pulverization was conducted to obtain nanocrystals and then the zirconia milling beads were removed through a screen. The suspension was diluted by the addition of an aqueous solution. The particle size of each nTivo formulation was measured using dynamic light scattering.

### Animals

#### Rabbits

Kbl:Dutch rabbits (pigmented rabbits) aged 14 to 39 weeks (male) or 13 weeks (female) and female Kbl:New Zealand White (NZW) rabbits (albino rabbits) aged 11 weeks were obtained from Kitayama Labes Co., Ltd. (Nagano, Japan). The rabbits were individually housed in open cages and maintained on a 12:12-hour light:dark cycle with constant temperature (19–25°C) and humidity (30%–70%). Each cage was provided with unrestricted access to municipal water and provided sterilized food (LRC4, Oriental Yeast Co., Ltd. Tokyo, Japan) once daily. For environmental enrichment, toys and timothy hay were supplied. The rabbits were acclimated for at least 1 week before the start of the study.

#### Monkeys

Male and female cynomolgus monkeys (*Macaca fascicularis*, Tian Hu Primate Animal Breeding Research Center Ltd.) aged 2 to 4 years were used in this study. The monkeys were individually housed in cages and maintained on a 12:12-hour light:dark cycle with constant temperature (23–29°C) and humidity (30%–70%). Each cage was provided with unrestricted access to municipal water and sterilized food (HF Primate J 12G 5K9J; Purina Mills LLC, Gray Summit, MO, USA) once daily.

### Animal Experiments

All animal experiments were conducted in compliance with the Association for Research in Vision and Ophthalmology (ARVO) Statement for the Use of Animals in Ophthalmic and Vision Research. Studies conducted at Kyowa Kirin Co., Ltd. were approved by the Institutional Animal Care and Use Committee (IACUC), and Kyowa Kirin Co., Ltd. is accredited by the Association for Assessment and Accreditation of Laboratory Animal Care International (AAALAC) International. Studies conducted at Shin Nippon Biomedical Laboratories, Ltd. (SNBL) were approved by the SNBL IACUC, and SNBL was also accredited by the AAALAC International. Humane endpoints were predefined and approved by the respective IACUCs. Animals were monitored regularly for general health, and euthanasia at scheduled necropsy was performed according to institutional standard.

### Pharmacokinetic Studies in Rabbits

Experimental designs for pharmacokinetic studies in rabbits are presented in Table 1.

**Table 1.** Experimental Designs for Pharmacokinetic Studies of nTivo and Microcrystallized Tivozanib in Rabbits

Experimental Purpose/Figure No.	Test Article Formulations (Average Particle Size and Formulation Concentration)	Dose	Dosing Regimen	Animals	Collected Samples	Sample Collection Times
Drug delivery efficiency/Figure 1	nTivo^*^ (64.39 nm and 1.72 mg/mL; 138.8 nm and 2.02 mg/mL; 364.9 nm and 2.05 mg/mL)Microcrystallized tivozanib (7530 nm and 5.49 mg/mL)	0.034 to 0.041 mg/eye0.11 mg/eye	Single ocular instillation	Pigmented rabbits	Retina/choroid	1.5 h
Concentration-time profile and ocular tissue distribution/Figure 2	nTivo^*^ (204 nm and 10 mg/mL)	0.3 mg/eye	Single ocular instillationMultiple ocular instillations (once a day for 14 d)	Pigmented rabbits	Cornea, conjunctiva, aqueous humor, vitreous body, sclera, iris/ciliary body, retina/choroid, serum	0.25, 1, 2, 4, 8, 24, 72 h0.25, 1, 2, 4, 8, 24, 72, and 168 h after the 14th dosing
Concentration-time profile and melanin-binding/Figure 3	nTivo^*^ (177.7 nm and 5 mg/mL)nTivo^*^ (179.3 nm and 5 mg/mL)	0.1 mg/eye	Single ocular instillation	Pigmented rabbitsAlbino rabbits	Retina/choroid, plasma	0.5, 1.5, 4, 7, 24 h0.25, 0.5, 1.5, 4, 7, 24 h

* Nanocrystallized tivozanib.

#### Test Article Formulations

Either the nTivo eye drop formulation or the microcrystal formulation was used. The average particle size and formulation concentration are provided in Table 1.

#### Dosing

Details of the dose and dosing regimen are provided in Table 1. The nTivo eye drops were ocularly instilled to one eye of each pigmented rabbit by instilling 20 or 30 µL onto the middle of the cornea using a micropipette. After dosing, the eyes were kept open for approximately 60 seconds to prevent spillage. In studies using albino rabbits nTivo eye drops (20 µL) were dropped slowly into the conjunctival sac, and the upper and lower eyelids were gently held together for 60 seconds. In pigmented rabbits, it has been confirmed that different instillation methods (with eyes open or closed for instillation) do not result in significant differences in exposure in the retina and choroid or systemic circulation (data not shown).

#### Sample Collection

After inducing anesthesia by an intravenous injection of thiopental sodium solution (1.2–1.5 mL/kg) into the auricular vein, blood samples were collected from the abdominal vena cava into polypropylene tubes. The serum samples were collected by centrifuging blood samples (room temperature, 1700×*g*, 5 minutes) after storage at room temperature for 20 to 60 minutes. The plasma samples were collected by centrifuging blood samples (4°C, 1700 or 8000×*g*, 10 minutes). The serum and plasma samples were stored at ≤−70°C for further processing and analysis. Prior to ocular tissue collection, animals were euthanized by exsanguination. Approximately 0.1 mL of aqueous humor was collected by inserting a needle attached to a disposable syringe containing 100 µL acetonitrile into the eyeball. The retina and choroid (hereafter: retina/choroid) were collected together from each rabbit. The ocular tissues (cornea, conjunctiva, aqueous humor, vitreous body, sclera, iris/ciliary body, and retina/choroid) were stored at ≤−70°C for further processing and analysis.

#### Sample Preparation

All ocular tissues except aqueous humor and vitreous body were weighed and nine times their weight in dilution solvent (acetonitrile and physiological saline mixed in ratio of 50:50 [v/v] or 50 vol% methanol) was added (based on the assumption that the specific gravity of ocular tissue is 1 [1 mg = 1 µL]). Samples were then homogenized using a multi-beads shocker, TissueLyser II (QIAGEN), or bead-impact type cell disruption device (ShakeMaster Auto; Medical Bio Sciences Co., Ltd.). The vitreous body was homogenized without dilution solvent. Serum, plasma, aqueous humor samples, and ocular tissue homogenate samples were precipitated with ice-cold acetonitrile containing an internal standard. After centrifugation, the supernatants were mixed with 5 mmol/L ammonium acetate aqueous solution.

#### Quantitative Measurement of Tivozanib Concentration

Typical measurement methods are briefly described below. The tivozanib concentration in serum or ocular tissues was measured using liquid chromatography coupled with tandem mass spectrometry (LC-MS/MS) consisting of the Shimadzu 20A system (Shimadzu Corporation, Kyoto, Japan) and API4000 mass spectrometer (SCIEX, Framingham, MA, USA). Prepared samples were separated by YMC Pack Pro C-18 S-5 (5-µm particle size, 2.0 × 50 mm; YMC Co., Ltd., Kyoto, Japan) with mobile phase A (5 mmol/L ammonium acetate aqueous solution) and mobile phase B (acetonitrile) at a flow rate of 0.2 or 0.4 mL/min. The LC gradient program was set for ocular tissue samples as follows: mobile phase B, initially setting 40%, then gradually increased to 75% for 2 minutes (from 0.00 to 2.00 minutes), and further increased to 90% for 0.5 minutes (from 2.01 to 2.50 minutes), then decreased to 40% and held for 1.5 minutes (from 2.51 to 4.00 minutes). Multiple reaction monitoring was performed using an electrospray ionization turbo source with positive mode, and monitor ion of tivozanib, KRN633 (internal standard), and ^13^C, ^2^H-labeled tivozanib (internal standard) were 455/357 (*m*/*z*, Q1/Q3), 417/358 (*m*/*z*, Q1/Q3), and 459/361 (*m*/*z*, Q1/Q3), respectively. Analyst (version 1.6.1, SCIEX) was used for the acquisition and analysis of LC-MS/MS data. The lower limit of quantification was 0.1 ng/mL for serum and 0.1 ng/g tissue for ocular tissues.

#### Pharmacokinetic Analysis

The mean tivozanib concentration-time profiles of serum, plasma, or ocular tissues were used for the pharmacokinetic analysis. A non-compartmental analysis was performed using Phoenix WinNonlin (version 8.1. or 8.5; Certara, Radnor, PA, USA), using the linear trapezoidal linear interpolation method. The pharmacokinetic parameters included the following: maximum concentration (C_max_), time to reach the maximum concentration (T_max_), area under the concentration-time curve (AUC) from zero to the last time point with measurable concentration (AUC_0–t_), AUC from zero to infinity (AUC_0–∞_), and elimination half-life (t_1/2_). The t_1/2_ was calculated using the Best Fit method of Phoenix WinNonlin, except for t_1/2_ in the retina/choroid in pigmented rabbits after a single ocular instillation, which was calculated within a specified time range of 4 to 72 hours post-dosing (Table 2).

**Table 2.** Pharmacokinetic Parameters of Tivozanib in the Retina/Choroid and Serum After Single and Repeated (Once a Day for 14 Days) Ocular Instillation of nTivo (0.3 mg/eye) to Pigmented Rabbits

					AUC_0–t_	AUC_0–∞_
PK Parameter	Tissue	C_max_ ng/g	T_max_ h	t_1/2_ h	ng·h/g	ng·h/g
Single ocular instillation	Retina/choroid	191	8.0	36.4	5,840^†^	8,200
	Serum	83.5^*^	1.0	4.7	541^†^^,^^‡^	552^‡^
Repeated ocular instillation	Retina/choroid	497	4.0	247.5	26,600^§^	68,700
	Serum	89.3^*^	4.0	49.7	688^‡^^,^^§^	731^‡^

The mean tivozanib concentration (*n* = 3 at each time point)-time profiles of the retina/choroid or serum was used for the pharmacokinetic analysis.

* Unit = ng/mL.

† AUC_0–72h_.

‡ Unit = ng·h/mL.

§ AUC_0–168h_.

### Laser-Induced Choroidal Neovascularization Cynomolgus Monkey Model

The experimental design for the pharmacological evaluation study using monkey CNV models is described below.

#### Induction of CNV

Thirty male cynomolgus monkeys (*Macaca fascicularis*) were included in the study. After confirming the dilation of the pupil after instillation of a mydriatic (Mydrin-P ophthalmic solution; Santen Pharmaceutical Co., Ltd.), animals were anesthetized by intramuscular injection (0.2 mL/kg) with a mixed solution of ketamine hydrochloride (Arevipharma GmbH, 50 mg/mL) and xyladine (20 mg/mL, Selactar 2% injection solution; Bayer Yakuhin, Ltd.; 7:1 [v/v]). A fundus contact lens (Centralis Direct; Volk Optical Inc.) was attached to the cornea with a special corneal protective coupling agent (Scopisol solution; Senju Pharmaceutical Co., Ltd.), and the position of the macula was confirmed. A 532-nm green laser was applied to the macula, avoiding the fovea, to create eight laser spots with a Multicolor Scan Laser Photocoagulator (MC-500, NIDEK Co., Ltd.). The laser (power = 1000 mW, spot size = 80 µm, and duration = 0.1 seconds) was applied to both eyes. Then, a topical antibiotic agent (Cravit ophthalmic solution 0.5%; Santen Pharmaceutical Co., Ltd.) was instilled into one eye once daily for 3 days. For each monkey, the one eye with confirmed CNV induction was treated with the test article.

#### Test Article Formulation and Dosing

The nTivo eye drops (average particle size = 169 nm, 5 mg/mL, 30 µL/eye) were ocularly instilled once weekly (0.15 mg/eye/week) for 3 weeks, or once (0.15 mg/eye/day) or 3 times (0.45 mg/eye/day) daily for 3 weeks from 3 weeks after laser injury. Intravitreal injections of aflibercept (50 µL/eye, 0.5 mg/eye) were performed in the laser-treated eye using a 30-gauge needle attached to disposable syringe under anesthesia by intramuscular injection (0.2 mL/kg, 10 mg/kg) with ketamine hydrochloride (Arevipharma GmbH, 50 mg/mL). The injection was performed once, 3 weeks after laser injury. After injection, a topical antibiotic agent (Cravit ophthalmic solution 0.5%; Santen Pharmaceutical Co., Ltd.) was instilled into the eye once daily for 3 days from the day after administration. Each group consisted of six monkeys.

#### Sample Collection, Sample Preparation, and Measurement of Tivozanib Concentration

Ocular tissues (choroid and retina) were collected on the day after the end of the administration period (day 22). Sample preparation and measurement of tivozanib concentrations were performed using the same methods as in the rabbit pharmacokinetic study with the following exceptions. The LC-MS/MS system consisted of an Agilent 1200 (Agilent Technologies, Inc., Santa Clara, CA, USA), HTC PAL (CTC Analytics AG, Zwingen, Switzerland), and API5000 (SCIEX) with atmospheric pressure chemical ionization mode. The LC gradient program was modified slightly. The lower limit of quantification was 10 ng/g tissue.

#### Image Analysis of Fluorescein Angiography Leakage

The development of active CNV lesions was assessed using fluorescein angiography (FA), conducted once prior to the first dosing and 8 and 21 days post-dosing. Examinations were performed under mydriasis and anesthesia by gross macroscopic examination and ophthalmoscopy. Fluorescein (fluorescent fundus contrast agent, Fluorescite intravenous injection 500 mg; Alcon Japan Ltd., 0.1 mL/kg, 0.1 mL/s) was administered into the cephalic vein on the forearm with a disposable syringe and indwelling needle. Photographs of the ocular fundus were taken approximately 5 minutes after fluorescein administration using an ocular fundus camera (VX-10α; Kowa Co., Ltd.). The fluorescein angiogram images were digitized using AnalySIS (Soft Imaging System GmbH) and the area of CNV was analyzed. For quantitative analysis, FA images obtained approximately 5 minutes after fluorescein injection were converted to 8-bit grayscale and intensity-inverted prior to analysis. The optic nerve head was manually delineated as an internal reference region of interest for threshold determination and area normalization. Fluorescent areas corresponding to CNV lesions were quantified as pixel counts after binarization and normalized to the optic nerve head area within the same image. Relative changes were calculated as percentages relative to pre-dosing values using standardized, predefined procedures.

### Local Ocular Safety Evaluation in Rabbits and Cynomolgus Monkey

#### Rabbits

The nTivo eye drops (average particle size = 187 nm, 20 mg/mL, 10 µL/eye/time) were administered by ocular instillation 10 times at 30-minute intervals to Japanese white rabbits (3 males). The anterior ocular segment and corneal epithelia (with fluorescein staining) were examined using a slit lamp up to 72 hours after the final dose and evaluated using the McDonald-Shadduck method.^29^ The middle optic media and ocular fundus were also examined using a slit lamp and indirect ophthalmoscope, respectively, after the instillation of the mydriatic agent. After completion of the examination, the animals were anesthetized by an intravenous injection of thiopental sodium solution (25 mg/mL, 1.3–1.5 mL/kg) and euthanized by exsanguination. Both eyeballs (including the optic nerves and bulbar conjunctiva) and eyelids were examined histopathologically.

#### Monkeys

The nTivo eye drops (average particle size = 169 nm, 5, 10 and 20 mg/mL, 30 µL/eye/time) were administered via ocular instillation to the left eye of cynomolgus monkeys (5 males and 5 females per group) 6 times daily at approximately 1-hour intervals for 4 weeks to evaluate eye toxicity. At the end of the administration period, intraocular pressure (IOP) was evaluated using a TonoVet Tonometer. In accordance with criteria for the evaluation using the McDonald-Shadduck method,^29^ gross observations and pupillary light reflex examinations were performed using a portable slit lamp. For all examinations except for pupillary light reflex and iris examinations, after instillation of a mydriatic, animals were anesthetized by an intramuscular injection (0.2 mL/kg, 10 mg/kg) of ketamine hydrochloride. To examine impaired corneal epithelia, fluorescein staining was evaluated using a portable slit lamp. After the gross and slit lamp examinations, ocular fundus was examined using an indirect ophthalmoscope. After ocular fundus examination, a surface anesthetic for ophthalmology was instilled into the eyes under anesthesia, and corneal thickness was examined using a corneal thickness measurement device. After completion of the examination, the animals were anesthetized by an intravenous injection of sodium pentobarbital (64.8 mg/mL, 0.4 mL/kg) and euthanized by exsanguination. Both eyeballs (including optic nerves and bulbar conjunctiva) and both eyelids were examined histopathologically.

#### Statistical Analysis

For the calculation of significant differences in the experiment measuring changes in the area of CNV lesion, a Williams test was conducted using SAS (version 9.4; SAS Institute Inc., Cary, NC, USA). *P* values of < 0.05 were considered statistically significant.

## Results

### Drug Delivery Efficiency of nTivo Eye Drops and Conventional Tivozanib Eye Drop Formulations With Microcrystals to the Retina/Choroid in Pigmented Rabbits

**Figure 1.**
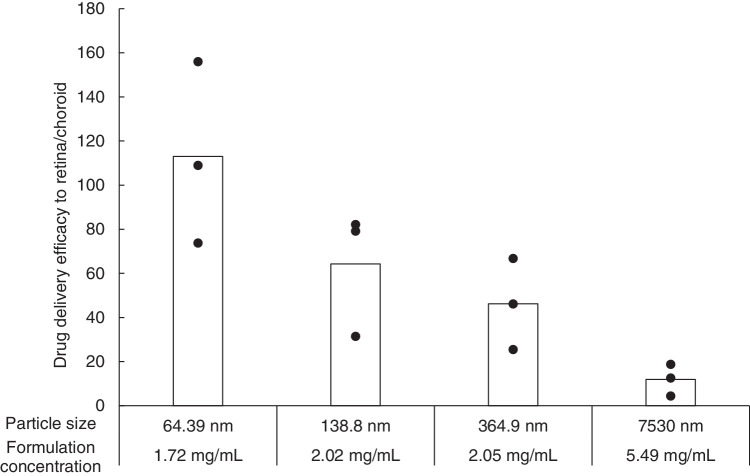
**Drug delivery efficacy of tivozanib to the retina and choroid after a single ocular instillation of nanocrystal and microcrystal formulations of tivozanib to pigmented rabbits.** Four different formulations of tivozanib with different average particle sizes and concentrations (64.39 nm and 1.72 mg/mL; 138.8 nm and 2.02 mg/mL; 364.9 nm and 2.05 mg/mL; and 7530 nm and 5.49 mg/mL) were administered to one eye of each rabbit (20 µL/eye) by a single ocular instillation. The tivozanib concentrations in the retina and choroid at 1.5 hours post-dose were measured. The drug delivery efficiency was calculated by dividing the tivozanib concentration in the retina and choroid by the formulation concentration. Each plot represents the drug delivery efficiency to the retina and choroid. The bar graph represents the mean of three animals.

We investigated the influence of particle size on the delivery efficiency of tivozanib to the retina/choroid in pigmented rabbits. The tivozanib concentration in the retina/choroid at 1.5 hours post-dose was measured. To compare different formulations, the drug delivery efficiency to the retina/choroid was calculated by dividing the tivozanib concentration in the retina/choroid by the formulation concentration. Figure 1 demonstrates that the delivery efficiency of tivozanib to the retina/choroid increased as the particle size decreased, reaching up to 9.5 times higher in the 64.39-nm nanocrystal formulation relative to the 7530-nm microcrystal formulation.

### Tivozanib Concentration-Time Profile in Ocular Tissues and Serum in Pigmented Rabbits

**Figure 2.**
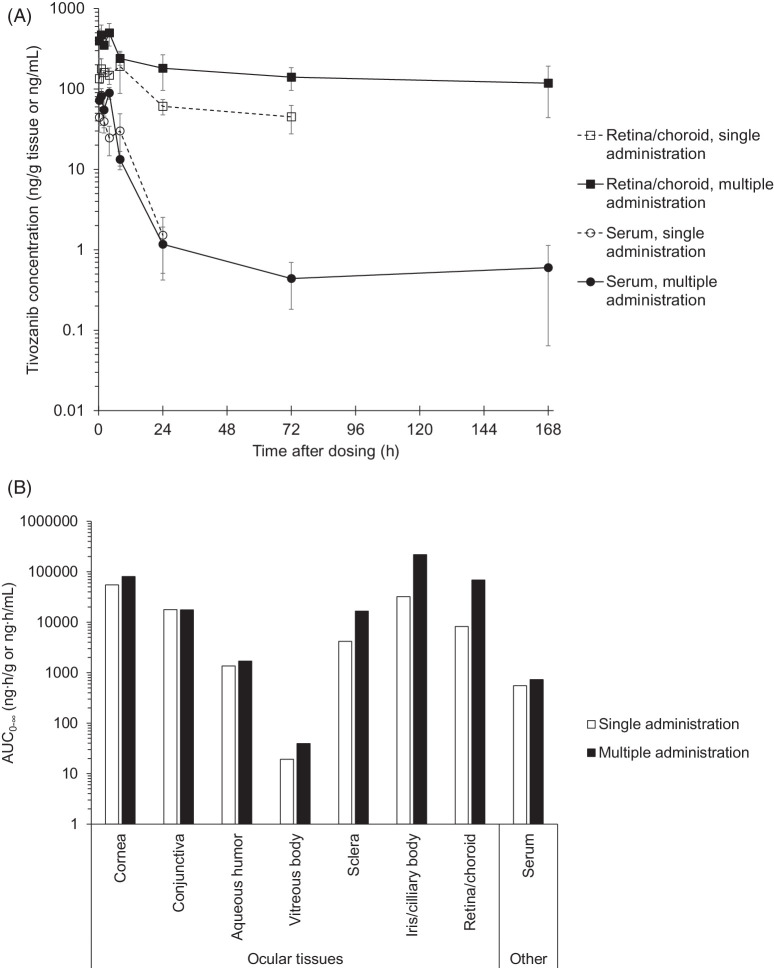
**Tivozanib concentration-time profile in the retina/choroid and serum, and exposure in ocular tissues and serum after single and multiple ocular instillations of nTivo to pigmented rabbits.** The nTivo at 0.3 mg/eye (10 mg/mL formulation, 30 µL/eye) was administered to one eye of Dutch (pigmented) rabbits by a single ocular instillation or multiple ocular instillations once a day for 14 consecutive days. At 0 (only for multiple administration), 0.25, 1, 2, 4, 8, 24, 72, and 168 (only for multiple administration) hours post-dosing, tivozanib concentrations in the retina/choroid and serum were measured. (**A**) Tivozanib concentration-time profile in the retina/choroid and serum. The lower limit of quantification was 0.1 ng/g tissue for the retina/choroid and 0.1 ng/mL for serum. Each plot and bar represent the mean ± standard deviation of three animals. *Black squares* = tivozanib concentrations in the retina/choroid after nTivo administration for 14 days. *White squares* = tivozanib concentrations in the retina/choroid after a single nTivo administration. *Black circles* = tivozanib concentrations in serum after nTivo administration for 14 days. *White circles* = tivozanib concentrations in serum after a single nTivo administration. (**B**) Tivozanib exposure in ocular tissues and serum. *White bars* = tivozanib exposure in 7 ocular tissues and serum after a single nTivo administration. *Black bars* = tivozanib exposure in 7 ocular tissues and serum after nTivo administration for 14 days.

Tivozanib concentration-time profiles after single or multiple (once daily for 14 days) ocular instillations of nTivo at 0.3 mg/eye (10 mg/mL formulation, 30 µL/eye) to pigmented rabbits were investigated. After a single ocular instillation, a consistently higher tivozanib concentration was observed in the retina/choroid relative to serum (Fig. 2A). Multiple ocular instillations of nTivo resulted in an 8.4-fold increase of tivozanib exposure as AUC_0–∞_ in the retina/choroid relative to a single administration, whereas tivozanib exposure in the serum was comparable between single and multiple instillations. The estimated t_1/2_ after 14 days of multiple ocular instillations was 247.5 hours in the retina/choroid and was longer than that observed in the serum (49.7 hours; see Table 2). The distribution of tivozanib to ocular tissues other than the retina/choroid was also evaluated (Fig. 2B). Whereas relatively higher exposure was observed in the anterior eye segment (cornea and conjunctiva) relative to the posterior segment (retina/choroid), the exposure of tivozanib was detected in aqueous humor and vitreous body, albeit at low levels. The exposure of tivozanib was also observed in the sclera and iris/ciliary body. In melanin-containing tissues (iris/ciliary body and retina/choroid), the exposure increased approximately seven- to eight-fold with multiple instillations.

### Tivozanib Concentration-Time Profile in the Retina/Choroid and Plasma in Pigmented or Albino Rabbits

**Figure 3.**
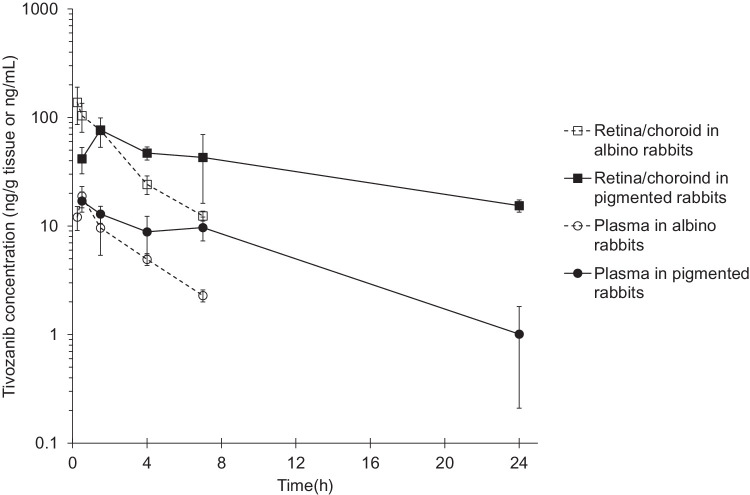
**Tivozanib concentration-time profile in the retina/choroid and plasma after a single ocular instillation of nTivo to pigmented or albino rabbits.** The nTivo at 0.1 mg/eye (5 mg/mL formulation, 20 µL/eye) was ocularly instilled to one eye of Dutch (pigmented) rabbits or NZW (albino) rabbits. At 0.25 (only for albino rabbits), 0.5, 1.5, 4, 7, and 24 hours post-dosing, tivozanib concentrations in the retina/choroid and plasma were measured. Each plot and bar represent the mean ± standard deviation of three animals. The lower limit of quantification was 1 ng/g tissue for the retina/choroid and 0.1 ng/mL for plasma. *White squares* = tivozanib concentrations in the retina/choroid in albino rabbits. *Black squares* = tivozanib concentrations in the retina/choroid in pigmented rabbits. *White circles* = tivozanib concentrations in plasma in albino rabbits. *Black circles* = tivozanib concentrations in plasma in pigmented rabbits.

Tivozanib showed melanin binding with longer t_1/2_ of radioactivity in the choroid layer of the eye than in all other tissues in a pigmented rat tissue distribution study using radio-labeled tivozanib.^27^ Therefore, to investigate the influence of the melanin-binding property of tivozanib on the pharmacokinetics in the posterior eye segment, we compared the tissue and plasma concentration-time profiles of tivozanib after a single ocular instillation of nTivo at 0.1 mg/eye in pigmented and albino rabbits (Fig. 3; Table 3). In albino rabbits, the tivozanib concentration in the retina/choroid rapidly reached C_max_ at 0.25 hours post-dose and its concentration-time profile showed a t_1/2_ of 2.1 hours, which closely paralleled that in plasma (t_1/2_, 2.7 hours). In contrast, in pigmented rabbits, the C_max_ of tivozanib in the retina/choroid was observed at 1.5 hours post-dose, with an approximately 2-fold difference in t_1/2_ (plasma = 5.9 hours, retina/choroid = 12 hours). The pigmented rabbits showed 5.7-fold longer t_1/2_ and a higher exposure (3.2-fold as AUC_0–∞_) in the retina/choroid relative to albino rabbits. These findings suggest that the melanin-binding property of tivozanib contributes to the accumulation and retention of tivozanib in the retina/choroid in pigmented rabbits.

**Table 3.** Pharmacokinetic Parameters of Tivozanib in the Retina/Choroid and Plasma After a Single Ocular Instillation of nTivo at 0.1 mg/eye to Albino and Pigmented Rabbits

		C_max_	T_max_	t_1/2_	AUC_0–t_	AUC_0–∞_
PK Parameter	Tissue	ng/g	h	h	ng·h/g	ng·h/g
Albino rabbits	Retina/choroid	138	0.25	2.1	318^†^	351
	Plasma	18.9^*^	0.5	2.7	48.7^†^^,^^‡^	57.4^‡^
Pigmented rabbits	Retina/choroid	76.7	1.5	12	855^§^	1120
	Plasma	17.0^*^	0.5	5.9	165^‡^^,^^§^	174^‡^

The mean the tivozanib concentration (*n* = 3 at each time point)-time profiles of the retina/choroid or plasma was used for the pharmacokinetic analysis.

* Unit = ng/mL.

† AUC_0–7h_.

‡ Unit = ng·h/mL.

§ AUC_0–24h_.

### Anti-Angiogenic Efficacy of nTivo Eye Drops in the Monkey CNV Model

The anti-angiogenic efficacy of nTivo eye drops on laser-induced CNV were evaluated in cynomolgus monkeys. The nTivo eye drops were ocularly instilled once weekly (0.15 mg/eye/week), once daily (0.15 mg/eye/day), or 3 times daily (0.45 mg/eye/day) for 3 weeks. Vehicle was ocularly instilled as a negative control 3 times a day for 3 weeks, and the positive control group received a single intravitreal administration of aflibercept (0.5 mg/eye) on day 1. Figure 4A illustrates the schedule of dosing and FA following the day of laser induction.

**Figure 4.**
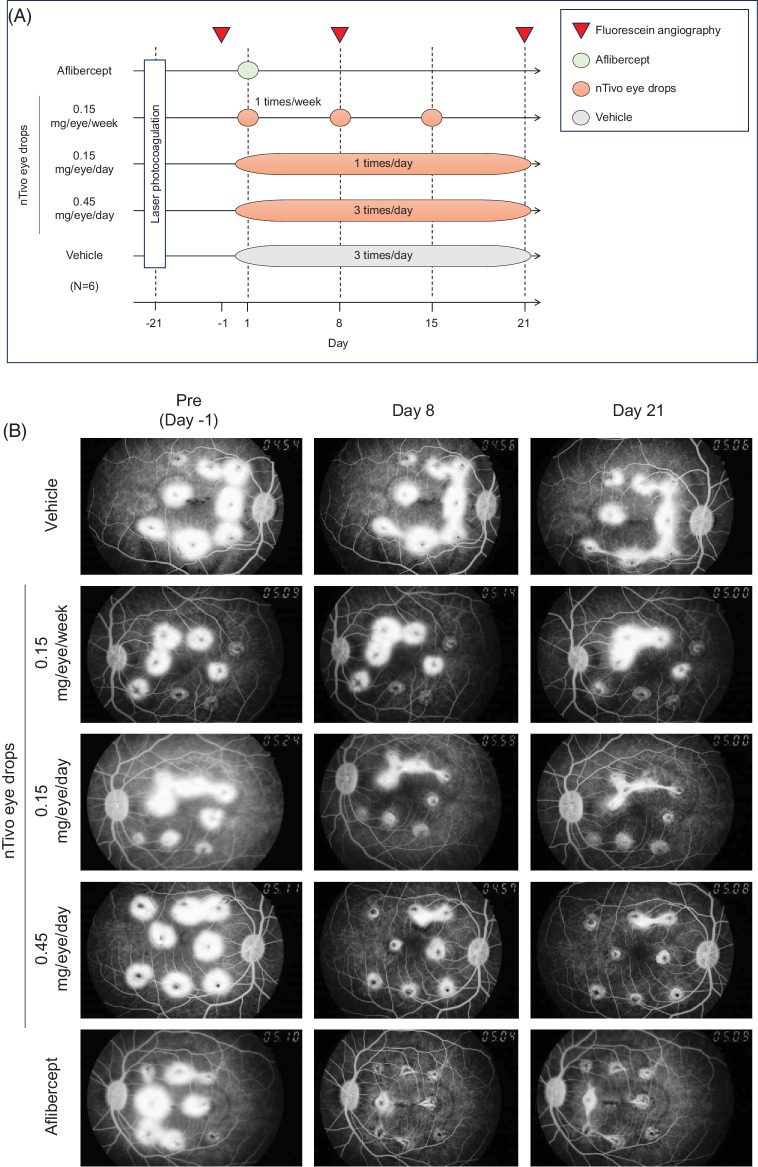
**Change of the area of laser-induced CNV lesions from pre-dosing in cynomolgus monkeys.** The nTivo eye drops were administered once weekly (0.15 mg/eye/week), once daily (0.15 mg/eye/day) or 3 times daily (0.45 mg/eye/day) for 3 weeks, starting 3 weeks after laser injury. The negative control group received vehicle 3 times daily for 3 weeks. The positive control group received a single intravitreal injection of aflibercept (0.5 mg/eye) on day 1. Choroidal neovascularization lesions were evaluated using fluorescein angiography 1 day before the first administration and at 8 and 21 days after the first administration. (**A**) Timeline for dosing and fluorescein angiography relative to laser photocoagulation. (**B**) Representative fluorescein angiography images one day before the first administration (day –1) and at day 8 and day 21 after the first administration. (**C**) The change in the area of CNV lesion. The values and vertical bar represent the mean ± SE of six monkeys. (**D**) The area of CNV lesion at day 21. Data are presented as box-and-whisker plots, where the boxes indicate the interquartile range (IQR) with the median shown as a *horizontal line*, and the whiskers represent the minimum and maximum values (excluding outliers). *Circles* represent outliers beyond 1.5 × IQR (*n* = 6 monkeys). *P < 0.05 versus negative control group by Williams test among the negative control and nTivo eye drop (0.15 mg/eye/week, 0.15 mg/eye/day, and 0.45 mg/eye/day) groups.

**Figure 4.**
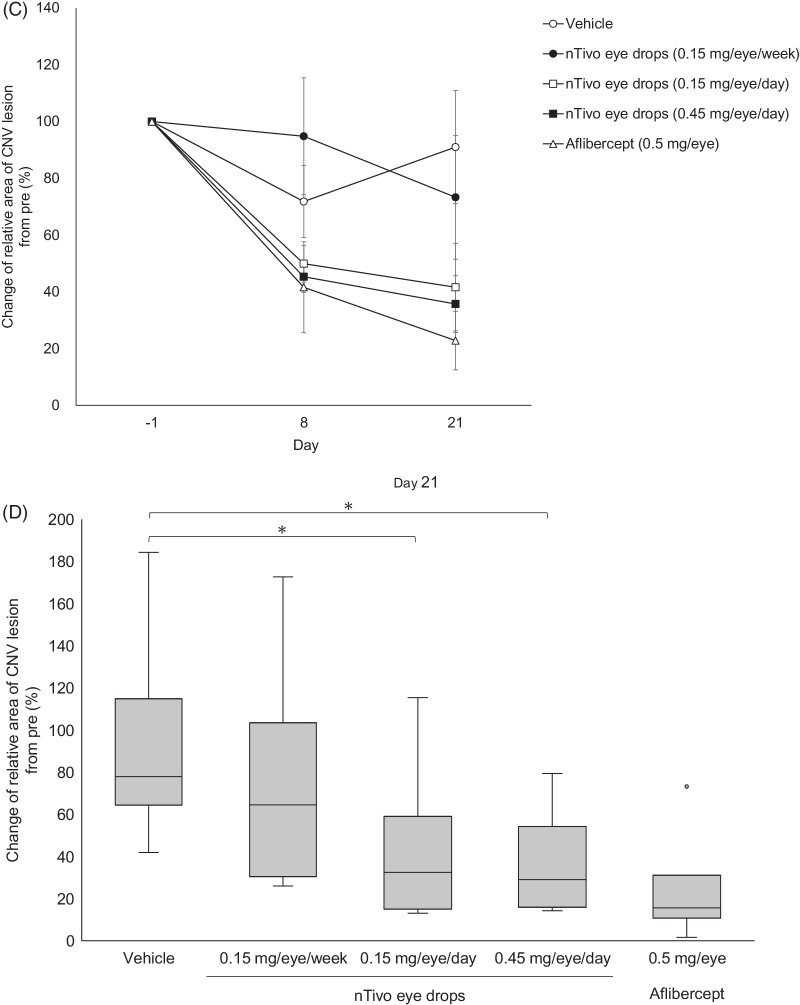
Continued.

The CNV lesions in the treated eye were evaluated by FA 1 day prior to the first administration and at 8 and 21 days post-administration. Dose-dependent anti-angiogenic efficacy was observed in the nTivo eye drop-treated groups relative to the vehicle-treated group, with the inhibitory effect observed at day 8 and maintained through day 21 (Figs. 4B, 4C). The anti-angiogenic efficacy was statistically significant in both nTivo (0.15 mg/eye/day) and nTivo (0.45 mg/eye/day) eye drop groups in comparison to the vehicle-treated group (negative control), whereas the nTivo (0.15 mg/eye/week) eye drop group did not differ significantly from the vehicle-treated group (Fig. 4D). Furthermore, tivozanib concentrations in the choroid and retina were measured on day 22, after completing the administration period. Tivozanib concentrations in both the choroid and retina increased with increasing dose levels (Table 4). No test article‑related changes were observed in any animals in ocular fundus, gross, or slit‑lamp examinations, indicating the absence of detectable ocular structural abnormalities.

**Table 4.** Tivozanib Concentrations in the Choroid and Retina on the Day After the End of 3-Week Period of Repeated Ocular Instillation of nTivo in a Cynomolgus Monkey CNV Model

		Tivozanib Concentration, ng/g	
Daily Dose, mg/Eye/Day	Dose Frequency	Choroid	Retina
0.15^*^	Once/week	39.0 ± 9.4	BLQ
0.15	Once/day	363 ± 97	27.9 ± 16.2
0.45	3 times/day	1400 ± 570	91.9 ± 43.9

BLQ, below the lower limit of quantification (<10 ng/g tissue).

Mean ± standard deviation (*n* = 6).

* Instilled on days 1, 8, and 15.

### Local Ocular Safety Evaluation in Rabbits and Cynomolgus Monkeys

In rabbits, slight eye irritation (conjunctival congestion and conjunctival discharge) was observed at 1 hour after the final dosing, however, these irritations resolved within 24 hours after the final dosing. No other test article-related abnormalities were observed in the corneal epithelia (fluorescein staining and histopathological examination) and the retina in any animal treated with nTivo eye drops. In monkeys, no test article-related changes were noted in any group at the end of the administration period.

## Discussion

In this study, the influence of particle size on the delivery efficiency of tivozanib to the retina/choroid was investigated in pigmented rabbits. Tivozanib delivery efficiency to the retina/choroid increased as particle size decreased, and the 64.39-nm formulation demonstrated a 9.5-fold increase in delivery efficiency compared to the 7530-nm formulation. After multiple ocular instillations of nTivo eye drops to pigmented rabbits, tivozanib exposure in the retina/choroid was significantly higher in comparison to a single ocular instillation. Relatively higher exposure was observed in the anterior eye segment (cornea and conjunctiva) relative to the posterior segment (retina/choroid). Tivozanib exposure was also observed in the sclera and iris/ciliary body. In melanin-containing tissues (iris/ciliary body and retina/choroid), the exposure increased approximately seven- to eight-fold with multiple ocular instillations. The exposure in the serum was comparable between single and multiple ocular instillations. Furthermore, a single ocular instillation of nTivo to both albino and pigmented rabbits revealed approximately six-fold longer t_1/2_ and three-fold higher exposure in the retina/choroid in pigmented rabbits. These results suggest that the enhanced ocular delivery achieved through nanocrystallization, along with the intrinsic melanin-binding properties of tivozanib, may contribute to the elevated drug exposure levels observed in posterior eye tissues. These two mechanisms of action of nTivo eye drops may contribute to the anti-angiogenic efficacy of nTivo eye drops in the monkey CNV model.

Previous studies have indicated that nanocrystallization can alter the physicochemical properties of poorly soluble drugs.^30^ By increasing the drug surface area and enhancing the dissolution pressure of strongly curved nanocrystals, nanocrystallized drugs can improve saturation solubility and the dissolution rate.^30^ Given that tivozanib itself exhibits poor solubility in water, with a reported value of 0.09 mg/mL,^31^ nanocrystallization could potentially enhance its saturation solubility and dissolution speed, thereby increasing its bioavailability from the eye. A limited portion of topical eye drops can reach posterior segments of the eye through anatomic and physiological barriers. The primary routes through which eye drops can be delivered, are corneal, conjunctival–scleral, and conjunctival–systemic, each with inherent limitations.^32^ The results of ocular tissue distribution in Figure 2B suggest that tivozanib distributes from the anterior to the posterior eye segment primarily via the corneal or conjunctival–scleral route following instillation. Focusing on the conjunctival sac, which faces the cornea and conjunctiva, the conjunctival sac typically holds approximately 7 µL of tear fluid, with an estimated turnover rate of 16% per minute.^33^ The calculated turnover time for complete tear-fluid replacement is approximately 6 minutes. Furthermore, the maximum capacity of the conjunctival sac is approximately 30 µL, and excess solution typically drains out through the nasolacrimal duct or overflows from the eye.^34^ Thus, increasing the saturation solubility of a drug to create a high-concentration eye drop formulation may also serve as a reasonable strategy to facilitate drug delivery to the eye.

Pazopanib (a VEGFR inhibitor) eye drops, failed to demonstrate sufficient efficacy in clinical trials for nAMD, which ultimately led to the termination of the study.^18^^,^^19^ One possible explanation for this lack of efficacy may be related to inadequate drug delivery to the posterior segment of the eye, as suggested by a previous report showing that pazopanib did not exhibit therapeutic effects in a cynomolgus monkey CNV model (a representative pathophysiological model of nAMD).^25^ In contrast, the present study demonstrated the significant anti-angiogenic efficacy of nTivo eye drops in the same laser-induced monkey CNV model. At an effective dose of 0.15 mg/eye/day, the concentration of tivozanib in the choroid was approximately 363 ng/g tissue, which is more than 10-fold higher than the concentration of pazopanib (18.3 ng/g) measured at the same dose.^25^ Furthermore, the half-maximal inhibitory concentration (IC_50_) value for VEGFR2 of tivozanib (0.07 ng/mL)^26^ is lower than that of pazopanib (3 ng/mL).^35^ Therefore, the posterior segment concentration of tivozanib was confirmed to reach levels substantially higher than its IC_50_ value, and this level was greater than that observed for pazopanib at the same dose. These findings suggest that the effective delivery of therapeutic agents to the target posterior eye tissues is likely important for achieving an effective therapeutic effect in the CNV model. Taken together, nTivo eye drops could be a potential therapeutic agent for treating posterior eye diseases, such as nAMD.

This study had several limitations regarding the extrapolation of findings from animals to humans. Even though nTivo showed preferable pharmacokinetic profile and anti-angiogenic efficacy in animals, it is well-known that there are large interspecies differences between animals and humans in ocular pharmacokinetics and anti-angiogenic efficacy after ocular instillation of eye drops. For example, although rabbits are commonly used for the evaluation of ocular pharmacokinetics of eye drops because the rabbit eye size is similar to the human eye, the blinking frequency of rabbits is lower than that of humans, and the nictitating membrane in the rabbit eye does not exist in the human eye.^36^ With respect to monkeys, whereas the monkey’s ocular structure is much more similar to the human ocular structure, the vitreous body volume is approximately half that of humans.^36^^,^^37^ Species differences in melanin content have also been reported among species.^38^ In our study, delivery of the tivozanib to the retina and choroid was observed in rabbits and monkeys, however, considering species differences in the structural anatomy of the eye or melanin content in ocular tissues, further studies are needed to elucidate the factors influencing drug delivery to the posterior segment of the eye in order to accurately predict the posterior delivery mechanism in humans.

Beyond interspecies differences, this study had several limitations specifically related to posterior-segment delivery. First, although tivozanib exposure was confirmed in the retina and choroid following ocular instillation, these measurements were based on tissue homogenates and therefore reflect drug levels at the tissue level rather than spatially resolved distribution within the posterior segment. As such, a detailed assessment of regional distribution within ocular tissues remains to be further investigated. Second, although ocular distribution data suggest that tivozanib reaches the posterior segment via corneal and/or conjunctival–scleral routes, the relative contribution of each pathway could not be quantitatively determined, and distribution along the outer ocular tissues may have contributed to the observed exposure. Third, tivozanib exhibits melanin-binding properties, as suggested by the prolonged half-life and higher exposure observed in pigmented rabbits, a phenomenon consistent with previous reports for topically administered melanin-binding drugs, such as atropine,^39^ pilocarpine,^39^ and levofloxacin.^40^ Whereas melanin binding may function as a depot and prolong ocular retention, it has also been reported that high melanin binding can reduce the unbound, pharmacologically active fraction of the drug at the target site, potentially attenuating pharmacodynamic effects.^39^^,^^40^ In the present study, unbound tivozanib concentrations were not measured, nor was the pharmacological activity of melanin-bound tivozanib evaluated in vitro; therefore, the relationship among melanin binding, local free drug exposure, and therapeutic efficacy remains to be elucidated. Finally, efficacy in the monkey CNV model was primarily assessed using FA, and additional structural and functional endpoints, such as optical coherence tomography (OCT)-based metrics and histological analyses, were not included.

For ocular safety, slight and reversible eye irritation (conjunctival congestion and discharge) was observed when nTivo eye drops were ocularly instilled to rabbits. In monkeys, no test article-related irritation was noted. Comprehensively, nTivo did not show severe ocular toxicity.

## Conclusions

This study demonstrates the preferable ocular pharmacokinetic characteristics and anti-angiogenic activity of nTivo eye drops in preclinical models potentially supported by nanocrystallization and melanin-binding properties of tivozanib, suggesting that nTivo eye drops could have a potential to be a new treatment option in nAMD.
